# Pharmacological and Therapeutic Potential of *Chrysopogon zizanioides* (Vetiver): A Comprehensive Review of Its Medicinal Applications and Future Prospects

**DOI:** 10.3390/biom15091312

**Published:** 2025-09-12

**Authors:** Conjeevaram J. Gunasekar, Amin F. Majdalawieh, Imad A. Abu-Yousef, Sham A. Al Refaai

**Affiliations:** 1 Loyola College, Madras University, Chennai 600034, Tamil Nadu, India; drsekhar62@gmail.com; 2 Department of Biology, Chemistry and Environmental Sciences, College of Arts and Sciences, American University of Sharjah, Sharjah P.O. Box 26666, United Arab Emirates; iabuyousef@aus.edu (I.A.A.-Y.); g00092818@alumni.aus.edu (S.A.A.R.); 3 Advanced Biosciences and Bioengineering Research Center, American University of Sharjah, Sharjah P.O. Box 26666, United Arab Emirates

**Keywords:** *C. zizanioides*, vetiver, anticancer, anti-inflammatory, analgesic, antioxidant, antimicrobial

## Abstract

*Chrysopogon zizanioides* (Linn.) Nash, commonly known as vetiver, has been an integral component of traditional medicinal systems across India and Asia for centuries. The roots and essential oils of this aromatic grass have been widely utilized for their anti-inflammatory, analgesic, anticancer, antioxidant, antimicrobial, and wound-healing properties. Recent scientific investigations have provided substantial evidence supporting these traditional claims, revealing a diverse array of bioactive phytochemicals with significant pharmacological potential. Preclinical studies have demonstrated the efficacy of *C. zizanioides* extracts in mitigating inflammation, alleviating pain, combating microbial infections, and even exhibiting anticancer and antidiabetic effects. This review provides a comprehensive analysis of the current literature on the therapeutic properties of *C. zizanioides*, summarizing findings from in vitro assays, cell line studies, animal models, and available clinical studies. The bioactive constituents responsible for these pharmacological effects, including essential oil components and isolated fractions, are discussed, along with their proposed mechanisms of action. These mechanisms involve modulation of oxidative stress, inflammatory pathways, microbial proliferation, and pain perception. Additionally, current research limitations, gaps in knowledge, and future directions for investigating medicinal applications of *C. zizanioides* are explored. Emerging scientific evidence increasingly validates traditional claims regarding the healing properties of this versatile medicinal grass.

## 1. Introduction

*Chrysopogon zizanioides* (Linn.) Nash, commonly known as vetiver, is a densely tufted, perennial grass belonging to the Poaceae family. Native to India, it is widely distributed across tropical and subtropical regions of South and Southeast Asia, including Malaysia, Sri Lanka, the Philippines, Pakistan, and Thailand [1]. The plant is primarily valued for its fragrant root system, which produces essential oils and bioactive compounds with diverse medicinal applications [1]. *C. zizanioides* has been extensively utilized in traditional Indian medical systems such as Unani, Siddha, and Ayurveda for centuries. In Sanskrit, it is referred to as *Ushira* and is known for its cooling, astringent, and therapeutic properties [2]. Historically, it has been employed in the treatment of heat stroke, inflammation, headaches, digestive disorders, skin conditions, and wound healing [1,2]. Ancient medical texts, including the Ayurveda and ancient Tamil literature, documented its medicinal use for treating fever, rheumatism, and urinary tract infections [3]. These historical applications suggest a broad pharmacological potential, which has been recently explored through scientific research.

Recent pharmacological research has provided substantial scientific validation for many of *C. zizanioides*’ traditional medicinal applications, linking its therapeutic efficacy to its diverse bioactive constituents. Extracts from *C. zizanioides* roots and essential oils have exhibited a broad spectrum of biological activities, including antimicrobial, antioxidant, anti-inflammatory, analgesic, and anticancer properties, in both in vitro and in vivo models [4]. The complexity of *C. zizanioides* essential oil is remarkable, with over 300 identified compounds, among which sesquiterpenes, flavonoids, and phenolic derivatives are the most biologically active [5]. Notably, key sesquiterpenoids such as cedr-8-en-13-ol display strong antimicrobial properties [6], while tannins display antifungal activity [7], and phenolic compounds contribute significantly to its anti-inflammatory effects [8]. As a result, *C. zizanioides*-based therapeutic applications have garnered increasing scientific interest and have been extensively explored for their potential in managing inflammatory disorders, infections, cancer, oxidative stress, and metabolic diseases such as diabetes and hypertension [9].

Despite promising preclinical findings, significant gaps remain in understanding the pharmacokinetics, bioavailability, and molecular mechanisms of action associated with *C. zizanioides*. Current research is limited by the lack of clinical trials to substantiate its therapeutic efficacy. This review provides a comprehensive synthesis of existing research on the bioactive compounds and pharmacological effects of *C. zizanioides*, integrating in vitro, in vivo, and clinical studies. Additionally, this review aims to explore proposed mechanisms of action, safety profiles, and key research gaps to guide future studies aimed at its development. Despite promising preclinical findings, significant gaps remain in understanding the pharmacokinetics and bioavailability of *C. zizanioides* and the molecular mechanisms underlying its medicinal applications.

## 2. Anti-Inflammatory Effects

Inflammation is a complex immunological response to harmful stimuli, including tissue injury, pathogen invasion, or environmental stressors [10]. While acute inflammation is an essential protective mechanism, chronic inflammation is implicated in the pathogenesis of numerous diseases, such as diabetes, arthritis, atherosclerosis, and neurodegenerative disorders [10,11]. Given the increasing prevalence of inflammatory diseases worldwide, natural sources of anti-inflammatory agents have garnered significant scientific interest [12,13,14]. *C. zizanioides* has been studied for such properties. However, most evidence available so far remains at the in vitro or animal model stage, and clinical trials in humans are currently lacking. Preclinical studies indicate that extracts and phytochemical fractions derived from *C. zizanioides* exhibit significant anti-inflammatory properties [15]. Both essential oils and solvent-based extracts, such as ethanolic and aqueous formulations, have demonstrated the ability to downregulate inflammation in cell culture assays and animal models [8,16]. These extracts effectively decrease the expression of key inflammatory cytokines and mediators, including tumor necrosis factor alpha (TNFα), interleukin-1 (IL-1), interleukin-6 (IL-6), cyclooxygenase-2 (COX-2), and nitric oxide (NO), while also inhibiting nuclear factor kappa B (NF-κB), a critical regulator of inflammatory pathways [8,16,17]. A detailed overview of these studies is presented in Table 1, summarizing both the in vitro and in vivo evidence supporting the anti-inflammatory properties of *C. zizanioides*.

Several in vitro studies have provided strong evidence of these effects. A study evaluated the essential oil extracted from roots in lipopolysaccharide (LPS)-stimulated RAW 264.7 murine macrophages, identifying 25 chemical constituents, of which cedr-8-en-13-ol (12.4%), α-amorphene (7.80%), β-vatirenene (5.94%), and α-gurjunene (5.91%) predominated. At non-cytotoxic concentrations of 5–12.5 μg/mL, *C. zizanioides* essential oil markedly reduced nitric oxide (NO) production in a dose-dependent manner, with the highest concentration lowering NO to 26.5% of the level observed in unstimulated controls. This was accompanied by significant downregulation of inducible nitric oxide synthase (iNOS) to 67% and cyclooxygenase-2 (COX-2) mRNA expression to 75% of LPS-induced levels, as well as reductions in tumor necrosis factor alpha (TNF-α) by ≥23% and interleukin-1 beta (IL-1β) by up to 81%. The oil simultaneously upregulated heme oxygenase-1 (HO-1) mRNA by 10–25%, suppressed interferon beta (IFN-β) transcription, decreased superoxide anion generation by up to 20%, and lowered lipid peroxidation below baseline values of unstimulated macrophages, indicating an interplay between its antioxidant and anti-inflammatory effects [16]. Moreover, ethanol extracts of *C. zizanioides* were assessed using the albumin denaturation assay, a model relevant to chronic inflammatory pathologies such as rheumatoid arthritis. The extract, obtained through 48 h maceration in ethanol (1:10 g/mL) and containing sesquiterpene alcohols such as vetiverol and vetivones, inhibited protein denaturation in a concentration-dependent manner (0–240 μg/mL) with an IC_50_ of 157.63 ± 4.89 μg/mL, representing approximately 2.8-fold lower potency than diclofenac (IC_50_ = 55.76 ± 2.35 μg/mL). This inhibitory effect is consistent with prior observations that vetiver essential oil reduces fibroblast collagen III production and leukocyte migration, both of which contribute to its anti-inflammatory potential [19,20,21]. These findings, as outlined in Table 1, collectively indicate that *C. zizanioides* extracts effectively reduce pro-inflammatory cytokine expression, specifically TNFα and IL-1β, across various cell types, supporting its traditional medicinal use as an anti-inflammatory agent. However, it is important to note that these in vitro findings, while promising, are preliminary and require further validation using in vivo models and human studies to confirm their physiological relevance.

Animal model studies further validate the anti-inflammatory efficacy of *C. zizanioides.* Methanolic root extract administered orally at 300 or 600 mg/kg significantly inhibited carrageenan-induced paw edema in Wistar rats, with the higher dose achieving 66.17% inhibition at 6 h post-injection, closely matching indomethacin’s 72.30% inhibition rate. In a cotton pellet-induced granuloma model, methanolic root extract reduced granuloma formation by 53.69%, suggesting suppression of fibroblast proliferation and extracellular matrix deposition in chronic inflammation [18]. Another study examined the root essential oil in carrageenan-induced peritonitis and paw edema models in mice. Intraperitoneal administration at 25–100 mg/kg reduced leukocyte migration by up to 62.5% and significantly inhibited all three inflammatory phases in paw edema swelling. In the formalin test, root essential oil suppressed the inflammatory (late) phase by 86% at 100 mg/kg without affecting the neurogenic (early) phase, indicating a peripheral site of action. Gas chromatography–mass spectrometry (GC-MS) analysis revealed major sesquiterpenes including khusimol, vetiselinenol, and cyclocopancamphan-12-ol. Notably, the root essential oil showed no significant activity in lipid peroxidation inhibition assays, suggesting its effects are not mediated by direct radical scavenging [22].

Chronic inflammation models further highlight *C. zizanioides*’ anti-inflammatory properties. A study compared ethanolic extracts from aerial parts (CA) and roots (CR) in a complete Freund’s adjuvant (CFA)-induced rheumatoid arthritis rat model. UHPLC/HRMS profiling identified CA as rich in phenolic compounds (42 identified, including flavonoid C-glycosides, lignans, and flavolignans), while CR contained fewer phenolics (13 identified) but higher levels of triterpenes and sesquiterpenes. Oral administration of both extracts significantly reduced serum anti-citrullinated protein antibodies (ACPA), IL-6, and TNF-α while restoring IL-10 levels, with CA exhibiting stronger modulation [8]. Molecular analyses revealed that both extracts downregulated Janus kinase 2 (JAK2) and signal transducer and activator of transcription 3 (STAT3) while upregulating suppressors of cytokine signaling 3 (SOCS3). Reductions in extracellular signal-regulated kinases 1/2 (ERK1/2) and TNF receptor-associated factor 6 (TRAF6) were also observed, alongside suppression of c-FOS, nuclear factor of activated T-cells cytoplasmic 1 (NFATC1), nuclear factor kappa B (NF-κB), and receptor activator of nuclear factor kappa-Β ligand (RANKL). Histopathological analysis confirmed marked attenuation of synovial inflammation, pannus formation, and cartilage destruction, particularly in CA-treated animals [8].

The mechanistic basis for these effects appears to involve a convergence of anti-inflammatory, antioxidant, and immunomodulatory actions. By downregulating NF-κB activation, through inhibition of nuclear translocation and DNA binding, *C. zizanioides* extracts reduce transcription of key inflammatory mediators such as TNF-α, IL-1β, IL-6, COX-2, and iNOS [8,16,23]. This modulation of transcriptional activity is reinforced by interference with upstream signaling pathways, including suppression of JAK2/STAT3, ERK1/2, and TRAF6, thereby attenuating both cytokine production and osteoclastogenesis [8,24,25,26]. Limitation of leukocyte recruitment to inflammatory sites, as shown in carrageenan-induced peritonitis [22] and further supported by reductions in chemokine-driven adhesion molecule expression [9], disrupts a critical step in the amplification of inflammation. Additionally, phenolic-rich extracts enhance cellular antioxidant defenses via increased activity of superoxide dismutase (SOD), glutathione peroxidase (GPX), and catalase (CAT), mitigating reactive oxygen species (ROS)-mediated activation of redox-sensitive transcription factors [9,16]. Certain constituents, including sesquiterpene alcohols such as khusimol and isovalencenol, further influence tissue remodeling processes by reducing fibroblast collagen III synthesis and limiting T-cell proliferation [9]. Flavonoids present in aerial extracts may also engage GABA-A receptors, producing central nervous system depressant effects that alleviate pain and stress associated with chronic inflammation [9,27,28].

**Figure 1:**
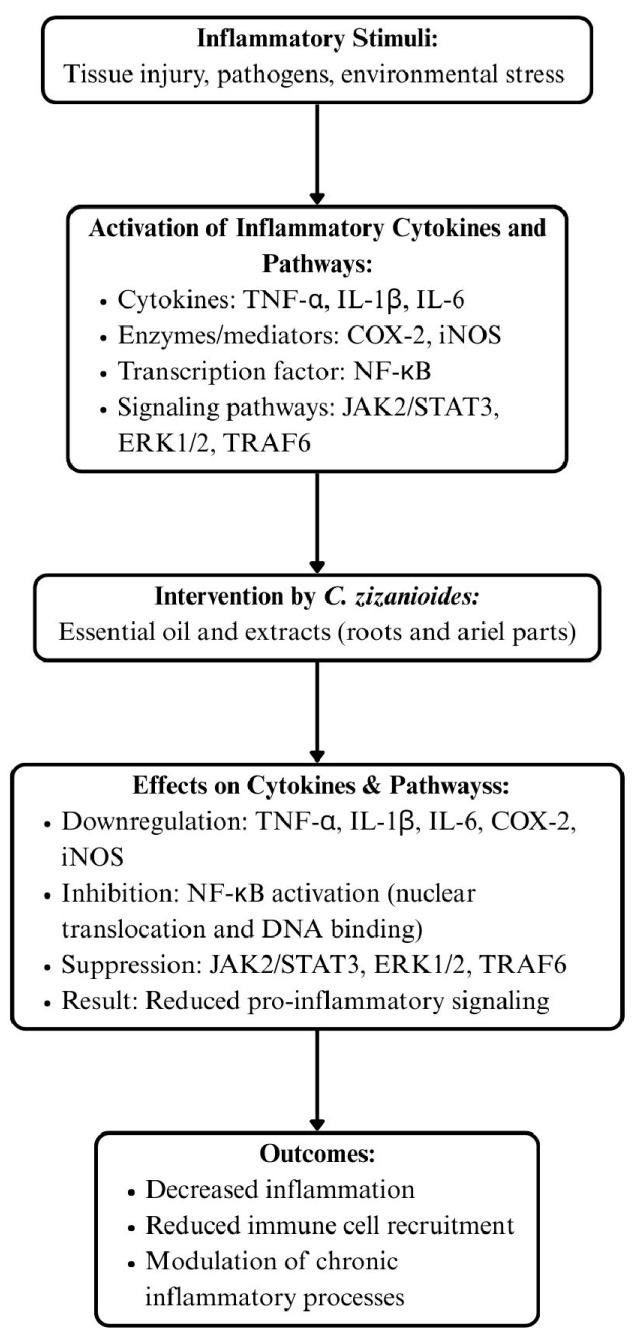
Cytokines and signaling pathways modulated by *C. zizanioides*.

Taken together, these studies indicate that *C. zizanioides* exerts multi-target anti-inflammatory effects through coordinated suppression of pro-inflammatory mediators, inhibition of immune cell infiltration, modulation of key intracellular signaling cascades, attenuation of oxidative stress, and regulation of tissue remodeling pathways. The differential efficacy of aerial versus root extracts highlights the importance of phytochemical composition in determining pharmacological activity, with phenolic-rich aerial parts and sesquiterpene-rich roots offering distinct yet complementary modes of action. While preclinical studies provide strong support for the anti-inflammatory potential of *C. zizanioides*, further research is necessary to explore its clinical relevance. Expanding on human clinical data, comparing its effects with existing anti-inflammatory drugs, and conducting dose–response and toxicity analyses will provide a more comprehensive evaluation of its therapeutic potential. Additionally, mechanistic studies investigating the interaction of its bioactive compounds with molecular targets could enhance our understanding of its pharmacological properties. A clearer understanding of pharmacokinetics, bioavailability, and safety profiles in human subjects should be explored to translate *C. zizanioides* from promising preclinical findings into clinical therapies, serving as a viable natural alternative for managing inflammatory disorders. The cytokines and signaling pathways modulated by *C. zizanioides* are summarized in Figure 1.

## 3. Analgesic Effects

Pain relief is a fundamental therapeutic goal in medical practice [29,30]. Conventional analgesic medications, such as opioids and nonsteroidal anti-inflammatory drugs (NSAIDs), are commonly used for pain management, but they are associated with adverse effects and the potential for abuse [31,32]. These concerns have fueled interest in alternative analgesics derived from natural sources, particularly medicinal plants. *C. zizanioides* has traditionally been employed for pain relief, and scientific investigations have begun to validate its ethnomedicinal application, revealing promising analgesic properties [33,34]. Analgesic effects of various *C. zizanioides* extracts have been demonstrated in a few in vitro studies. Studies have shown that both aqueous and alcoholic root extracts exhibit analgesic actions comparable to standard drugs like aspirin or morphine in tests such as acetic acid-induced writhing, hot plate, and tail immersion assays [22]. These results indicate significant attenuation of chemically and thermally induced nociceptive pain behaviors. Additionally, essential oil compositions containing *C. zizanioides* oils have displayed dose-dependent pain inhibition by lowering pain score parameters [22]. Research has explored the antinociceptive activity of *C. zizanioides* essential oil. A study by Lima et al. characterized *C. zizanioides* essential oil using GC/MS, identifying major constituents such as khusimol (19.57%), E-isovalencenol (13.24%), α-vetivone (5.25%), and β-vetivone (4.87%) [22]. Intraperitoneal administration of *C. zizanioides* essential oil at doses of 50 and 100 mg/kg significantly reduced the number of writhes by 51.9% and 64.9%, respectively, and decreased paw licking during the second phase of the formalin test by 56.7% and 86.2%, respectively, compared to control groups. However, the *C. zizanioides* essential oil did not exhibit significant effects in hot plate and rota-rod tests, suggesting a peripheral rather than central mechanism of action [22]. Another study on *C. zizanioides* leaf extract demonstrated 66.08% analgesic activity in animal models, compared to 91.11% for diclofenac sodium [35]. Collectively, these findings support the analgesic properties of *C. zizanioides*, reinforcing its traditional use in pain management. A detailed overview of these findings is presented in Table 2, highlighting the analgesic efficacy and experimental models used in recent studies. It is important to note that while these studies provide promising results, they stem from preclinical animal studies with limited sample sizes and variability in dosage, preparation, and administration routes. Therefore, the reproducibility and consistency of the findings are limited. As a result, there is a gap in the translation to human clinical therapies. More studies are warranted to ensure consistency in the results, and human clinical trials are crucial to strengthen the evidence supporting the analgesic efficacy of *C. zizanioides.*

The antinociceptive mechanisms of *C. zizanioides* are believed to involve multiple pathways, including modulation of inflammatory mediators, NO pathways, and possibly central pain signaling. *C. zizanioides* essential oil has been found to inhibit carrageenan-induced leukocyte migration and paw edema in rodents, suggesting suppression of key pain mediators such as prostaglandins. A study reported that *C. zizanioides* essential oil downregulates inducible nitric oxide synthase (iNOS) expression in macrophages, thereby reducing NO production, a pathway integral to pain modulation [22]. Although direct evidence of opioid receptor involvement remains limited, some essential oils with similar phytochemical compositions exert analgesic effects via opioid receptor interactions. One study found that *C. zizanioides* essential oil exhibited antinociceptive properties comparable to morphine in an acetic acid-induced writhing test. This effect was partially reversed by naloxone, an opioid receptor antagonist, indicating the potential involvement of opioid receptors in its analgesic action [22]. Additionally, *C. zizanioides* essential oil reduced pain behaviors in the second phase of the formalin test, associated with inflammatory pain, but did not show significant effects in the Hargreaves’ test, a measure of thermal nociception [22]. *C. zizanioides* shows the most promising therapeutic application in inflammatory and chemically induced pain models, with significant reductions in writhing and formalin test scores, while limited effects in thermal or central models suggest a targeted analgesic action. Moreover, neurotransmitter systems such as glutamate, GABA, and serotonin are critical in pain perception [36,37], and while studies specifically addressing *C. zizanioides* are scarce, findings from other essential oils suggest a possible role in modulating these pathways [9,38,39,40,41].

Phytochemical constituents such as monoterpenes, sesquiterpenes, and phenolic acids, which are found in *C. zizanioides* roots and essential oils, may contribute to these analgesic actions [22,35,42,43,44]. Despite promising activity in animal models, clinical evaluations of human efficacy and safety remain lacking. Future studies should focus on isolating bioactive compounds, understanding pharmacological interactions, conducting clinical trials on specific pain indications, and developing optimized drug delivery systems to enhance the therapeutic potential of *C. zizanioides*. Overall, *C. zizanioides* demonstrates notable analgesic potential through multiple molecular mechanisms, supporting its traditional use in pain relief. With future clinical research advancements, *C. zizanioides* holds promise as a safe, complementary analgesic targeting diverse pain pathways.

## 4. Antioxidant Properties

Antioxidant activity is a key therapeutic attribute of *C. zizanioides*, primarily mediated through its ability to neutralize free radicals and modulate endogenous antioxidant defense systems [35,45,46,47,48]. In vitro and in vivo studies have demonstrated that *C. zizanioides* extracts exhibit significant free radical scavenging, metal chelation, and enzymatic antioxidant modulation, all of which contribute to its protective effects against oxidative stress-related pathologies [16,49,50,51,52]. These diverse antioxidant mechanisms are summarized in Table 3, which outlines an overview of key studies and outcomes. Several in vitro assays, including DPPH, ABTS, and phosphomolybdenum, have been employed to evaluate these effects. Ethanolic, ethyl acetate, chloroform, ether, and aqueous extracts of the roots consistently display dose-dependent DPPH radical scavenging. Reported IC_50_ values span from as low as 10.73 µg/mL for ethanolic extract, comparable to ascorbic acid (4.61 µg/mL) [53], to higher values such as 157.38 µg/mL for ethanol and 112.79 µg/mL for ethyl acetate extracts [54]. Root essential oils show similar efficacy, producing 50% inhibition at concentrations around 184–185 µg/mL, with activity benchmarked against BHT and ascorbic acid [55]. The phytochemical composition of these extracts is central to their activity. Essential oils are particularly rich in sesquiterpenes such as longiverbenone, longipinocarvone, Cedr-8-en-13-ol, khusimol, α-vetivone, and bicyclo-vetivenol, whereas ethanolic extracts contain over a hundred compounds, including khusenic acid, ascorbic acid, junipen, γ-himachalene, and α-guaiene [35,53,54,55,56,57,58]. Phenolic acids, both soluble and bound forms, such as p-coumaric, p-dihydroxybenzoic, and ferulic acid, make important contributions through hydrogen donation and radical scavenging [56,57,59]. Flavonoids and other phenolic constituents further enhance activity by donating electrons and stabilizing reactive oxygen species [54,60]. Beyond radical scavenging, *C. zizanioides* extracts also exhibit ferric-reducing capacity. FRAP assays demonstrate that essential oils reduce Fe^3+^ to Fe^2+^ with EC_50_ values near 185 µg/mL, while phosphomolybdenum assays reveal total antioxidant capacities of up to 75.5% at 100 ppm, correlating closely with phenolic content [55,56]. These findings suggest that sesquiterpenes, terpenic alcohols, and phenolics act synergically, providing both radical-scavenging and electron-transfer mechanisms to suppress oxidative chain reactions.

Animal studies support the physiological relevance of these findings. In a murine model of acetaminophen-induced hepatotoxicity, crude aqueous root extracts restored hepatic glutathione (GSH) and the activities of glutathione reductase (GR) and glutathione S-transferase (GST) while normalizing GPx activity. Pre-treatment offered slightly greater protection than post-treatment. The extracts also reduced malondialdehyde (MDA) levels in liver tissue, indicating inhibition of lipid peroxidation and suggesting hepatoprotective effects mediated by phenolics and terpenic alcohols [58]. Studies on essential oils sourced from different regions, including Comoros and Egypt, show consistent dose-dependent radical scavenging [56,57]. Strong correlations between phenolic content and antioxidant activity in water-soluble fractions further highlight the pivotal role of phenolics in free radical neutralization [59].

Chemical profiling across investigations highlights the diversity of compounds responsible for these effects. Essential oils are dominated by sesquiterpenes, terpenic alcohols, and minor ketones, while ethanolic extracts are enriched with phenolics and flavonoids. This complex phytochemical composition enables synergistic interactions, combining hydrogen donation, electron transfer, and modulation of enzymatic defenses to produce strong antioxidant activity.

Despite the promising evidence, limitations exist. Most of the available evidence comes from preliminary in vitro studies or short-term in vivo models, leaving their direct applicability to human health uncertain. Differences in extraction methods, solvents, and assay conditions contribute to variability in reported IC_50_ and EC_50_ values. Moreover, the bioavailability, pharmacokinetics, and clinical efficacy of these compounds have not yet been clarified. While comparisons with reference antioxidants such as ascorbic acid and BHT are informative, they cannot fully predict performance in complex biological systems. Future work should therefore prioritize standardized extraction and testing protocols, detailed chemical profiling, and broader in vivo models. Greater attention should be given to synergistic interactions among bioactive compounds, dose–response dynamics, and long-term safety. Ultimately, clinical studies will be needed to validate the therapeutic potential of *C. zizanioides* as an antioxidant.

## 5. Antimicrobial Effects

The antibacterial properties of *C. zizanioides* have been investigated, demonstrating its efficacy against a broad range of pathogenic bacteria. These effects are typically assessed using methods such as minimum inhibitory concentration (MIC) assays, agar disk diffusion, and agar well diffusion to quantify antimicrobial effects, determine bacteriostatic versus bactericidal activity, and compare efficacy across microbial species [65,66,67,68]. In MIC assays, serial dilutions of *C. zizanioides* essential oils or crude extracts are incubated with bacterial cultures to identify the lowest concentration preventing visible growth, whereas diffusion-based approaches involve applying defined volumes of extract to agar plates and measuring inhibition zones after overnight incubation [65,66,69,70,71]. Although these assays provide a preliminary assessment, they do not account for pharmacokinetics, bioavailability, or host immune factors that affect clinical efficacy. Comparative analyses with standard antibiotics further substantiate the antimicrobial efficacy of *C. zizanioides* extracts, which have been shown to exhibit broad-spectrum activity against multidrug-resistant bacteria, viruses, and yeasts, reinforcing their traditional medicinal applications [69,70]. Recent studies further demonstrated strong inhibitory activity of *C. zizanioides* essential oil against methicillin-resistant Staphylococcus aureus (MRSA), with low MIC values, time-kill assays confirming bactericidal activity, and mechanistic evidence of protein, DNA, and RNA leakage due to membrane disruption [72,73]. Additionally, methanol and ethyl acetate extracts of *C. zizanioides* showed significant activity against Gram-negative bacteria such as *E. coli* and *P. mirabilis*, with inhibition zones ranging between 18 and 27 mm, and in polyherbal formulations, the activity extended synergistically to include *Pseudomonas aeruginosa* and *Enterobacter* sp. with inhibition zones up to 40 mm [74,75]. Commercial *C. zizanioides* oils have also been reported to be especially active against Gram-positive bacteria, including MRSA and Bacillus strains, while showing moderate antifungal activity against *Candida albicans* and strong inhibition of *Candida glabrata*, a clinically relevant azole-resistant pathogen [76,77].

Experimental studies consistently demonstrate that root-derived extracts and essential oils of *C. zizanioides* are particularly effective against Gram-positive bacteria such as *Staphylococcus aureus*, *Bacillus subtilis*, and *Bacillus cereus*, with inhibition zones ranging from 19.3 to 28.3 mm and MIC values as low as 0.0625% (*v*/*v*) for essential oils [65,69,70]. In contrast, Gram-negative bacteria, including *Escherichia coli* and *Pseudomonas aeruginosa*, generally exhibit reduced susceptibility, with inhibition zones as small as 9 mm, reflecting structural differences in cell envelopes [65,66]. Dose-dependent inhibition is observed in ethanolic and methanolic extracts, which display increasing activity at higher concentrations against *S. aureus*, *E. coli*, and *P. aeruginosa* [66,78]. Interestingly, *B. subtilis* shows minimal sensitivity to ethanolic extracts, illustrating species-specific variability in response to phytochemical constituents [66].

The antibacterial effects are largely attributed to the presence of diverse bioactive phytochemicals, including alkaloids, flavonoids, saponins, terpenoids, tannins, phenolics, and sesquiterpenes [65,66,79,80]. Essential oils, rich in cycloisolongifolene derivatives and other volatile compounds, act primarily by compromising bacterial cell membrane integrity, disrupting enzymatic activity, and interfering with metabolic pathways. The bactericidal potential is confirmed by MBC/MIC ratios ≤ 4 across tested strains, with Gram-positive bacteria consistently displaying the highest sensitivity [65]. A study reported that *C. zizanioides* exerts its antibacterial effect by disrupting bacterial membrane integrity, leading to the leakage of intracellular proteins, DNA, and RNA, with the effect being more pronounced at higher concentrations [73]. Comparisons with standard antibiotics reveal that *C. zizanioides* essential oils can achieve statistically significant inhibition relative to tetracycline and erythromycin, emphasizing their potency and wide-ranging antimicrobial capacity [65].

The antibacterial potential of *C. zizanioides* is particularly important when it comes to the efficacy against antibiotic-resistant bacteria. Hexane and ethanol extracts of *C. zizanioides* roots inhibit 55–70% of *methicillin-resistant S. aureus* (MRSA) isolates at remarkably low MIC values, with hexane extracts effective at 0.012 mg/mL. Such results underscore the potential of lipophilic constituents to overcome resistance mechanisms [72,80,81]. Furthermore, combinations of *C. zizanioides* essential oils with other plant oils, such as Pogostemon patchouli, demonstrate synergistic activity against MRSA, with ΣFIC indices ranging from 0.35 to 0.38, supporting the potential for combination therapies to enhance efficacy [82]. Innovative strategies leveraging nanotechnology have also been explored [83,84]. Silver nanoparticles synthesized using aqueous root extracts of *C. zizanioides* exhibit potent antibacterial effects against both *S. aureus* and *P. aeruginosa* bacteria. Nanoparticles, averaging 10–20 nm in diameter, are thought to act via enhanced surface interactions that compromise membrane integrity and enzymatic functions, illustrating a contemporary approach to improving pharmacodynamic properties of plant-derived antimicrobials [79,85].

While these are promising findings, it is important to note that certain methodological limitations are presented, such as variability in extract composition, concentration, and microbial strains tested. Moreover, few in vivo studies or clinical trials exist to confirm the antimicrobial efficacy and safety of *C. zizanioides* extracts in humans. Therefore, translation from in vitro results to clinical applications remains a critical gap. A detailed summary of the experimental assays and proposed mechanisms of action is provided in Table 4. Notwithstanding these limitations, variation in antimicrobial efficacy is influenced by extract type, solvent polarity, seasonal fluctuations in phytochemical content, and bacterial strain-specific susceptibility [65,66,78]. While in vitro studies demonstrate reproducible inhibitory effects, further research is required to assess pharmacokinetics, safety, and clinical applicability, alongside isolation and structural characterization of the active compounds responsible [65,66,79]. A detailed summary of the experimental assays and proposed mechanisms of action is provided in Table 4.

## 6. Wound Healing Potential

Skin health relies on a delicate balance of lipid metabolism, extracellular matrix regulation, and coordinated cellular signaling [86,87,88]. Natural bioactive compounds, particularly those derived from *C. zizanioides*, have recently attracted attention for their multifaceted roles in dermatology, ranging from anti-aging effects to tissue repair and remodeling. While current research is limited, preliminary results are promising. Tollenaere et al. (2021) demonstrated that *C. zizanioides* root extracts can stimulate lipid synthesis across multiple skin cell types, including keratinocytes, sebocytes, and adipocytes [89]. The extract not only increased sebum production in sebocytes by 31 percent but also improved lipid composition, elevating levels of lauric acid and sapienic acid by 42 and 43 percent, respectively [89]. Moreover, it enhanced epidermal barrier integrity through the upregulation of sphingomyelin, phosphoglycerides, ceramides, and cerebrosides, alongside key proteins involved in lipid transport and cornification, including CERT, involucrin, and dermokine. Importantly, *C. zizanioides* extract promoted both adipogenesis and lipogenesis in pre-adipocytes and full-thickness skin explants, resulting in a greater number and size of adipocytes [89]. Clinically, these effects translated into improved skin plumpness, reduced signs of fatigue, and a measurable decrease in wrinkle depth. Chemical analysis revealed a complex mixture of sesquiterpenes, including zizanoic acid, oplopanone, and teuhetenone, as well as several previously unreported compounds, underscoring the potential of these constituents as active agents in lipid regulation and skin rejuvenation [89]. In parallel, another study explored the effects of *C. zizanioides* essential oil in pre-inflamed human dermal fibroblasts, designed to model chronic inflammation and fibrosis. The study revealed that the essential oil exerted pronounced antiproliferative effects and significantly reduced collagen III production, a critical component of the extracellular matrix involved in tissue remodeling [90]. Genome-wide analyses showed that 163 genes were significantly regulated, most of which were associated with metabolic processes and tissue remodeling pathways, including cholesterol biosynthesis and inositol pyrophosphate metabolism [90]. These findings suggest that *C. zizanioides* essential oil may influence both structural and metabolic aspects of skin physiology, complementing the lipid-enhancing properties observed with *C. zizanioides* root extracts [90]. While no significant modulation of inflammatory biomarkers was observed in fibroblasts, previous studies in macrophages indicate that *C. zizanioides* essential oil possesses anti-inflammatory and antioxidant activities, highlighting potential cell-type-specific effects [16,22].

**Figure 2:**
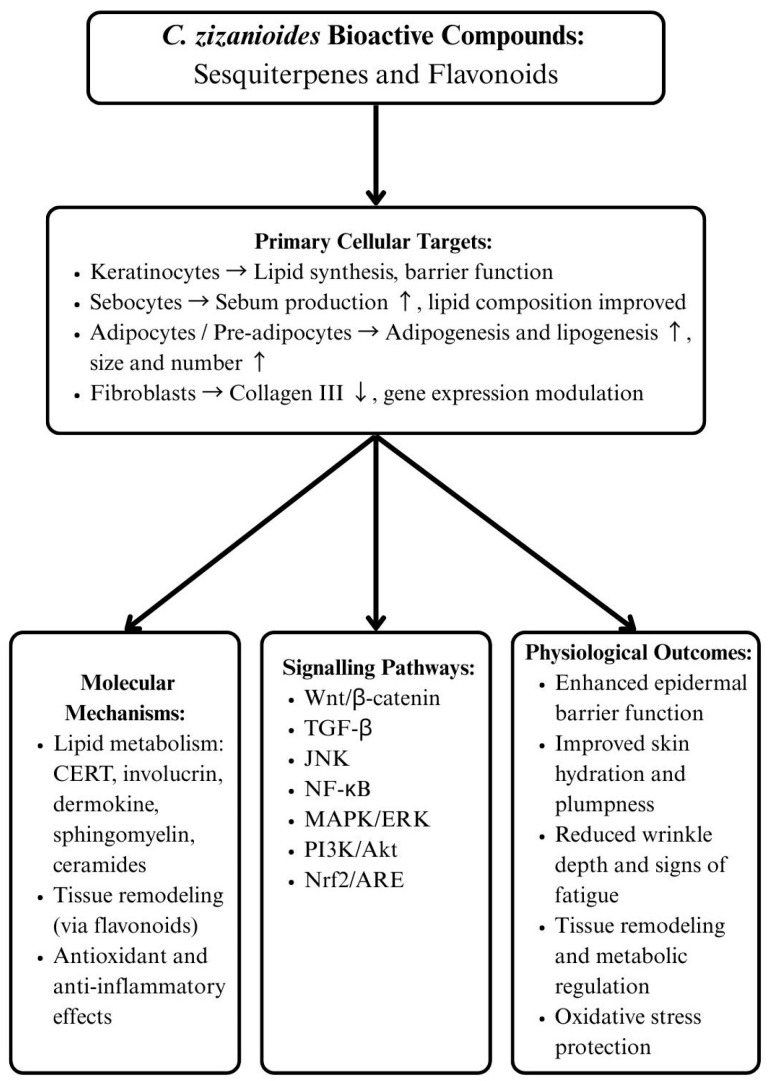
Wound healing potential of *C. zizanioides*. ↑ indicates an increase (upregulation), and ↓ indicates a decrease (downregulation) in the corresponding molecule or effect.

Beyond lipid metabolism and fibroblast activity, the broader molecular framework for skin repair involves a coordinated interplay of signaling pathways that govern inflammation, angiogenesis, re-epithelialization, and oxidative stress. Flavonoids promote wound healing through multiple pathways, including Wnt/β-catenin, Hippo, TGF-β, JNK, NF-κB, MAPK/ERK, PI3K/Akt, and Nrf2/ARE. These pathways collectively regulate cellular proliferation, differentiation, and antioxidant defenses [91]. Since, *C. zizanioides* extract have shown to be rich in flavonoids, it is possible that the mechanisms influenced by *C. zizanioides* extracts appear to converge with these pathways, as seen in Figure 2, particularly through modulation of lipid and cholesterol metabolism, suggesting a potential role in enhancing barrier function, tissue remodeling, and oxidative stress protection. However, these mechanisms should be studied further with *C. zizanioides* flavonoids rich extracts. Therefore, these studies highlight the potential of *C. zizanioides* derived compounds as multifunctional agents in skin health. Root extracts primarily support lipid synthesis, sebum quality, and adipocyte function, contributing to hydration, plumpness, and visible anti-aging effects. Essential oil influences fibroblast activity and gene expression, affecting tissue remodeling and metabolic regulation. When considered alongside the mechanistic insights from flavonoid-mediated wound healing, it becomes evident that *C. zizanioides* bioactive may act across multiple levels, including lipid metabolism, extracellular matrix remodeling, and oxidative stress regulation, to strengthen barrier integrity, enhance resilience, and support tissue repair. As highlighted in Table 5, these findings provide a strong foundation for further research into *C. zizanioides* extracts and essential oil as therapeutic agents in dermatology, with potential applications in anti-aging, metabolic skin support, and wound healing.

## 7. Anticancer Activity

*C. zizanioides* has demonstrated notable anticancer potential through a combination of cytotoxic, apoptotic, and ROS-mediated mechanisms, both in vitro and in vivo. In vitro studies on various cancer cell lines have consistently shown that *C. zizanioides* extracts and derived compounds exert selective cytotoxicity. For instance, hydrodistilled *C. zizanioides* oil exhibited moderate cytotoxic activity against human lung (A549) and hepatocellular carcinoma (HepG2) cell lines, with IC50 values of 32.2 µg/mL and 37.6 µg/mL, respectively [92]. Similarly, *C. zizanioides* oil was evaluated against colon (WiDr), triple-negative breast cancer (4T1), and estrogen receptor-positive breast cancer (T47D) cells. *C. zizanioides* oil induced cell cycle arrest, primarily in G2/M phase for WiDr and 4T1 cells, with IC50 values of 302 µg/mL, 60 µg/mL, and 112 µg/mL for WiDr, 4T1, and T47D cells, respectively, highlighting selective cytotoxicity toward TNBC cells [93]. Mitotic index analysis further confirmed that crude *C. zizanioides* oil from agricultural sources suppressed proliferation of HeLa cervical cancer cells more effectively than commercial essential oil, with MI reductions from 5.57% (control) to 1.70% [94].

Mechanistically, the anticancer activity of *C. zizanioides* is frequently linked to the induction of oxidative stress through increased reactive oxygen species (ROS) levels. *C. zizanioides* oil treatment elevated intracellular ROS in WiDr and T47D cells, correlating with apoptosis induction, while 4T1 cells underwent G2/M arrest without significant ROS increase, suggesting alternative mechanisms of cytotoxicity [93]. Sesquiterpene components such as β-caryophyllene, khusimol, and β-vetispirene have been identified as major contributors to these effects. Notably, β-vetispirene selectively inhibited AKR1C1, an enzyme overexpressed in lung cancer that maintains intracellular ROS below apoptotic thresholds, without affecting the highly homologous AKR1C2, thereby promoting ROS-mediated apoptosis [95]. The selective targeting of AKR1C1 highlights a genetic and molecular basis for the anticancer efficacy of specific *C. zizanioides* compounds. Furthermore, *C. zizanioides* oil’s cytotoxicity in 4T1 TNBC cells has been attributed to its agonistic interaction with the Cannabinoid CB2 receptor (CNR2), a gene highly expressed in these cells and associated with poor patient survival, while estrogen receptor-expressing T47D cells displayed less sensitivity due to lower receptor engagement [93].

Several studies have confirmed the low cytotoxicity of *C. zizanioides* on normal cells, including HEK 293 kidney cells and L929 fibroblasts, indicating a favorable therapeutic index for potential anticancer applications [96,97]. Cytotoxicity assays consistently showed cell viability above 75% at concentrations where anticancer activity was observed in tumor cells. These findings support the selective activity of bioactive sesquiterpenes and essential oil components against malignant cells. Although in vivo studies directly evaluating tumor suppression are limited, the immunomodulatory properties of valencene, a major fraction of *C. zizanioides* root extract, suggest potential indirect anticancer benefits through immune system enhancement. Valencene, a compound found in *C. zizanioides* extract, significantly increased phagocytic index, nitric oxide scavenging, and antibody production in Swiss albino mice, while reducing pro-inflammatory TNF-α production, suggesting a dual role in enhancing antitumor immunity while minimizing systemic inflammation [96]. These immune effects may complement direct cytotoxic mechanisms in vivo, although formal tumor xenograft studies remain to be conducted.

Collectively, the current literature, as outlined in Table 6, indicates that *C. zizanioides* exerts anticancer activity through multiple convergent mechanisms, including selective induction of apoptosis, ROS-mediated oxidative stress, cell cycle arrest, and immune modulation. The identification of genetic targets such as AKR1C1 and CNR2 highlights molecular pathways that may be exploited for targeted therapy. The selective cytotoxicity, low toxicity to normal cells, and molecular specificity highlight the therapeutic promise of *C. zizanioides* and its sesquiterpene constituents for future anticancer drug development. While these in vitro studies provide important mechanistic insights, their methodological limitation lies in the simplified cellular models, and they may not fully translate to the complex human physiological system. Therefore, clinical studies are essential when evaluating therapeutic potential.

## 8. Conclusions

The pharmacological potential of *C. zizanioides* has been extensively explored, validating its traditional ethnomedical applications through modern scientific investigations. Its anti-inflammatory activity involves modulation of cytokines and inflammatory mediators, while its strong antioxidant capacity stems from free radical scavenging, metal chelation, and enhancement of endogenous antioxidant enzymes. *C. zizanioides* also exhibits broad-spectrum antimicrobial effects, alongside selective anticancer properties characterized by apoptosis induction and tumor growth inhibition. Additionally, *C. zizanioides* supports effective wound healing through the stimulation of fibroblast activity and extracellular matrix. Therefore, these pharmacological activities position *C. zizanioides* as a promising candidate for therapeutic development across diverse areas such as managing inflammatory disorders, infections, cancer, and tissue repair. These effects are attributed to its diverse bioactive constituents, including sesquiterpenes, flavonoids, and steroids, which exert their therapeutic influence by modulating molecular pathways and immune responses. However, most of the current evidence is derived from in vitro and animal studies with limited clinical data to confirm efficacy and safety in humans. Moreover, the variability in extract preparation, dosage, and bioavailability presents challenges for standardization and reproducibility. To bridge these gaps, future research must focus on clinical trials, pharmacokinetics, and toxicological studies. Only then can the full therapeutic potential of *C. zizanioides* be realized and translated into clinical applications.

## 9. Future Directions

To advance the use and integration of *C. zizanioides* into modern pharmacotherapy, several key research areas must be addressed. Comprehensive phytochemical profiling is necessary to characterize the full spectrum of bioactive compounds and elucidate their synergistic interactions within biological pathways. Pharmacokinetic studies should be conducted to determine the bioavailability, metabolic fate, and systemic distribution of active molecules, thereby providing insights into their therapeutic efficacy and safety. Additionally, specificity and interaction potential with other therapeutic agents should be assessed to facilitate rational drug design. Improving delivery systems, particularly for hydrophobic bioactive constituents, remains a critical challenge, and novel formulation strategies should be explored to enhance solubility and bioavailability. Most importantly, rigorous, well-controlled clinical trials must be initiated to establish the safety, efficacy, and therapeutic applicability of *C. zizanioides* across various medical indications. As research continues to elucidate the pharmacological mechanisms and clinical benefits of *C. zizanioides*, its traditional medicinal use can be effectively translated into evidence-based modern medicine, expanding its role in contemporary healthcare.

## Figures and Tables

**Table 1:** Summary of the preclinical studies on the anti-inflammatory effects of *C. zizanioides*.

Study Design	Experimental Model	Extract Type and Bioactive Compounds Involved	Dosage	Main Observed Effects	References
In vitro	RAW 264.7 macrophages (LPS-induced)	Essential oil; major constituents: cedr-8-en-13-ol (12.4%), α-amorphene (7.8%), β-vatirenene (5.94%), α-gurjunene (5.91%)	5–12.5 μg/mL	NO ↓ (to 26.5% at 12.5 μg/mL), iNOS ↓ (~67%), COX-2 ↓ (~75%), TNF-α ↓ (≥23%), IL-1β ↓ (up to 81%), IFN-β ↓; HO-1 ↑ (10–25%); superoxide anion ↓ 12–20%; lipid peroxidation ↓; apoptosis ↓	[16]
In vivo	CFA-induced rheumatoid arthritis in Wistar albino rats	*C. zizanioides* aerial (CA, phenolic-rich: flavonoids, lignans, flavolignans) and root (CR, triterpene- and sesquiterpene-rich) ethanolic extracts	200 mg/kg	ACPA ↓, IL-6 ↓, TNF-α ↓, IL-10 ↑ (CA > CR); JAK2/STAT3 ↓, SOCs3 ↑; ERK1/ERK2 ↓; TRAF6/c-FOS/NFATC1 ↓ (CA stronger), NF-κB ↓; RANKL ↓; synovial inflammation, pannus formation, cartilage destruction ↓	[8]
In vivo	Carrageenan-induced paw edema and cotton pellet-induced granuloma in Wistar rats	Root methanol extract	300–600 mg/kg	Acute: paw edema ↓ 66.17% at 600 mg/kg (6 h); chronic: granuloma formation ↓ 53.69% at 600 mg/kg; dose-dependent inhibition of early (histamine, serotonin, kinins) and late (prostaglandin) mediators; fibroblast proliferation and collagen synthesis ↓	[18]
In vitro	Albumin denaturation assay	Root ethanol extract; bioactives: vetiverol, vetivones	0–240 µg/mL	Protein denaturation ↓ dose-dependently; IC50 = 157.63 µg/mL (~2.8 × less potent than diclofenac)	[19]

↑ indicates an increase (upregulation), and ↓ indicates a decrease (downregulation) in the corresponding molecule or effect.

**Table 2:** Summary of the analgesic effects of *C. zizanioides*.

Extract Type	Bioactive Compound Involved	Model/Test Used	Key Findings	Comparative Efficacy	References
Root essential oil	khusimol, E-isovalencenol, α-/β-vetivone	Acetic acid writhing test (50 and 100 mg/kg) Formalin test: Phase 2 licking Hot plate and rota-rod test Carrageenan-induced leukocyte migration (peritoneal) Paw edema	Writhes: At 50/100 mg/kg: writhes inhibited by 51.9% and 64.9% Formalin Phase 2 licking reduced by 56.7% and 86.2%. No effect in hot plate or rotarod tests. ↓ Inflammation (dose-dependent leukocyte inhibition and edema suppression)	Aspirin inhibited writhing ~75%; vetiver EO achieved ~65% inhibition. Demonstrated peripheral-only analgesic action.	[22]
Ethanolic leave extract	GC/MS profiled 63 compounds including esters, sesquiterpenes, alcohols, hydrocarbons. Major compounds: 9,19-Cyclolanostan-3-ol acetate (8.8%), 13-Docosenamide (8.35%), γ-Sitosterol (5.2%)	Acetic acid writhing (oral, 500 mg/kg)	~66.08% analgesic activity	Compared to 91.11% for diclofenac sodium.	[35]

↓ indicates a decrease (downregulation) in the corresponding molecule or effect.

**Table 3:** Summary of the mechanisms underlying the antioxidant activity of *C. zizanioides*.

Study Design	Extract Type and Bioactive Compounds Identified	Assay/Model	Key Findings	Mechanism	References
In vitro	Ethanolic leaf extract of *C. zizanoides*; 63 phytoconstituents identified including 9,19-Cyclolanostan-3-ol acetate (3β) and Phytol	DPPH radical scavenging assay	Moderate antioxidant activity (IC50 = 257.23 µg/mL) compared to ascorbic acid.	Antioxidant via radical scavenging and SOD, catalase, GPx	[35]
In vitro	Aqueous and ethanolic whole plant extracts; glycosides, carbohydrates, phenols, flavonoids, saponins, gums, mucilage	FRAP, Nitric oxide scavenging, Hydrogen peroxide scavenging, DPPH-RSA	Ethanolic extract showed higher antioxidant activity than aqueous; correlated with phenolic and flavonoid content.	Antioxidant activity via free radical scavenging and electron donation	[49]
In vitro	Crude oil; β-vetivenene, β-vetivone, α-vetivone, khusimol, bicyclovetivenol	DPPH radical scavenging, Fe^2+^ metal chelating	Strong DPPH scavenging (~93% at 10 µL/mL); weak metal chelation.	Free radical scavenging via terpenoid constituents	[45]
In vitro	Essential oil; cedr-8-en-13-ol, α-amorphene, β-vatirenene, α-gurjunene	LPS-stimulated RAW 264.7 macrophages; superoxide anion, MDA, SOD assays	↓ Superoxide anion (12–20%), ↓ MDA, ↓ NO, ↓ apoptosis.	Anti-inflammatory: ↓ HO-1, iNOS, COX-2, TNF-α, IL-1β, IFN-β; Antioxidant: ↓ oxidative stress and lipid peroxidation	[16]
In vitro	*C. zizanioides* oil; complex mixture of terpenoids	DPPH radical scavenging assay	Strong antioxidant activity (93% scavenging at 10 µL/mL), ~α-tocopherol, >BHT.	Free radical scavenging by components of *C. zizanioides* oil	[46]
In vitro	Hexane root extracts of two genotypes; phenolics and flavonoids	FRAP, DPPH, TAC, RP, TPC; oxidative stress in erythrocytes (H_2_O_2_, t-BHP)	KS1 genotype (spent root KSD) showed the highest antioxidant activity (FRAP, DPPH, TPC); protected GSH and ↓ MDA under H_2_O_2_ stress.	Free radical scavenging via phenolics; antioxidant protection of erythrocytes	[50]
In vitro	Root essential oil extracted via CXE, HD, IVD, and SFE; major components valerenol, valerenal, β-cadinene, β-vetivenene	DPPH radical scavenging	CXE oil showed moderate antioxidant activity (IC50 3.71 mg/mL).	Free radical scavenging via terpenoid constituents	[61]
In vitro	Root ethanolic extract; contains alkaloids, flavonoids, tannins, phenols, saponins, triterpenoids	Reducing power assay, superoxide anion scavenging, deoxyribose degradation, total antioxidant capacity, total phenolics and flavonoids	Dose-dependent strong antioxidant activity; superoxide IC50 130.36 µg/mL; high phenolic and flavonoid content.	Free radical scavenging; hydroxyl radical neutralization; lipid peroxidation inhibition	[62]
In vitro	Silver nanoparticles synthesized from aqueous extract of *Vetiveria zizanioides*	DPPH free radical scavenging assay	Dose-dependent antioxidant activity; max inhibition 72.4% at 50 µL; comparable to standard.	Free radical scavenging by nanoparticle-mediated electron donation	[63]
In vitro	Aqueous, methanolic, and ethanol root extracts; bioactive compounds include phenolics, flavonoids, alkaloids, saponins, tannins.	DPPH, FRAP, ABTS radical scavenging assays	Dose-dependent antioxidant activity; high phenolics correlated with strong scavenging; ABTS and FRAP confirm potent activity.	Free radical scavenging via electron donation and H-atom transfer by phenolics and flavonoids	[64]
In vitro	Water-soluble, glycoside, and cell wall-bound phenolic acids; major: p-coumaric, p-dihydroxybenzoic, ferulic acids	ABTS assay (TEAC)	Alkaline water-soluble (cell wall-bound) fraction: highest phenolics and antioxidant activity; strong correlation (r = 0.988) with TEAC.	Antioxidant via free radical scavenging by phenolic acids; higher lignin-bound phenolics ↑ stress mitigation	[59]
In vitro	Essential oil; major compounds: khusimol, isovalencenol, 2-isopropyl-5-methyl-9-methylene-bicyclo [4.4.0]decene, α-vetivol, beta-maalene, vetiselinenol, γ-selinenes, zizanol, khusiol, β-vatirenes	Phosphomolybdenum assay	Essential oil showed 75.5% total antioxidant capacity at 0.1 mg/mL.	Antioxidant activity likely mediated by phenolic and other bioactive constituents	[56]
In vitro	Essential oil; major compounds: Khusimol (25.60%), Bicyclo-vetivenol (11.47%), α-Vetivone (7.76%)	DPPH radical scavenging assay	Dose-dependent antioxidant activity; highest activity 60.43% at 0.5 mg/mL, lowest 52.74% at 0.03125 mg/mL; activity lower than BHT.	Antioxidant activity mainly from terpenic alcohols and phenolics; both major and minor constituents contribute	[57]
In vivo	Crude water extract (roots)	Biochemical assays for serum ALT, AST, ALP; liver antioxidant enzymes: GSH, GR, GST, GPx; lipid peroxidation (MDA)	Pre/post Vetiveria extract ↓ ALT, AST, ALP; ↑ GSH, GR, GST; ↓ lipid peroxidation; pre-treatment > post-treatment.	Protection via maintaining hepatic antioxidants, ↑ GR and GST, ↓ oxidative stress and lipid peroxidation; partially mimics NAC	[58]
In vitro	Essential oil; main components: longiverbenone (27.31%), longipinocarvone (26.88%), Cedr-8-en-13-ol (26.26%)	DPPH free radical scavenging assay, FRAP assay	Dose-dependent DPPH and FRAP activity; FRAP IC50 = 184.8 ± 1.02 μg/mL; activity linked to phenolics.	Antioxidant activity from high EO phenolics; electron donation and free radical neutralization	[55]
In vitro	Ethanol and ethyl acetate extracts; rich in flavonoids and phenolic compounds	DPPH free radical scavenging assay	Both extracts: dose-dependent scavenging; 140 µg/mL inhibition: ethanol 40.7%, ethyl acetate 59.3%; IC50: ethanol 157.38, ethyl acetate 112.79 µg/mL.	Antioxidant activity via H-donation by phenolics and flavonoids, quenching free radicals	[54]
In vitro	Ethanolic root extract; major compounds include Khusenic acid, Ascorbic acid, Junipen, gamma-Himachalene, alpha-Guaiene	DPPH radical scavenging assay	Strong antioxidant activity (IC50 10.73 μg/mL; ascorbic acid 4.61 μg/mL)	Antioxidant-mediated free radical scavenging contributes to cytotoxicity in cancer cells	[53]

↑ indicates an increase (upregulation), and ↓ indicates a decrease (downregulation) in the corresponding molecule or effect.

**Table 4:** Summary of the mechanisms underlying the antimicrobial activity of *C. zizanioides*.

Study Design/Assay	Extract Type and Bioactive Compounds Involved	Target Microorganism	Key Findings	Mechanism of Action	References
MIC assay	Ethanol, hexane, methanol extracts; Essential oil; Phenolic acids; Flavonoids; Terpenoids	*S. aureus*, *B. subtilis*, *MRSA*, *E. coli*, *P. aeruginosa*, *Candida* spp.	Low MIC values indicate strong potency Effective against drug-resistant pathogens Comparable to standard antibiotics	Disruption of bacterial cell walls and membranes; interference with metabolic enzymes Inhibition of efflux pumps and virulence factors Membrane permeability increase leading to leakage of DNA, RNA, proteins	[36,37,38,44,45,46,48,49,51]
Mechanistic Observations (descriptive studies)	Essential oils, methanol/ethanol extracts; Terpenoids; Flavonoids; Phenolic acids	Broad spectrum (Gram-positive, Gram-negative, fungi)	Disrupts biofilm formation Impairs protein synthesis, nucleic acid metabolism Inhibits pili/fimbriae Reduces toxin and enzyme production	Disrupts nutrient uptake Disrupts nutrient uptake Induces ROS accumulation Damages proteins, lipids, DNA Increases membrane permeability leading to cell death	[65,73,74,75,79]
Disk diffusion/ Well diffusion	Essential oils; Root, leaf, and methanol extracts; Terpenoids; Flavonoids; Phenolic acids	*S. aureus*, *MRSA strains*, *E. coli*, *K. pneumoniae*, *P. aeruginosa*, *Candida* spp.	Inhibition zones 12–35 mm Strong antibacterial and antifungal activity Effective against multidrug-resistant strains	Diffusion of active compounds into agar Direct interaction with microbial membranes Membrane disruption Cell content leakage Inhibition of microbial growth at the zone of contact	[73,74,75,76,77,81,82]
Nanoparticle-mediated assay	Silver nanoparticles synthesized using root aqueous extract; Phytochemicals	*S. aureus*, *P. aeruginosa*	Potent antibacterial effect; Effective at low concentration (25 μg/mL)	Alteration of bacterial cell membrane permeability Enzyme inhibition Increased ROS generation	[85]

**Table 5:** Summary of the mechanisms underlying the wound healing activity of *C. zizanioides*.

Extract Type	Target Cells and Pathways	Mechanisms of Action	Observed Effects	Clinical Implications	References
Root Extract	Keratinocytes, sebocytes, adipocytes, skin explants	- ↑ Lipid synthesis (lauric acid ↑ 42%, sapienic acid ↑ 43%) - ↑ Sebum production (+31%) - ↑ Barrier lipids (sphingomyelin, ceramides, cerebrosides) - ↑ Proteins (CERT, involucrin, dermokine) - ↑ Adipogenesis and lipogenesis	- Improved epidermal barrier - Greater number/size of adipocytes - Enhanced plumpness - ↓ Wrinkle depth, ↓ fatigue signs	Hydration, anti-aging, barrier strengthening	[89]
Essential Oil	Pre-inflamed fibroblasts	- ↓ Collagen III (extracellular matrix remodeling) - Antiproliferative effect - Regulation of 163 genes (cholesterol biosynthesis, inositol pyrophosphate metabolism)	- Modulation of fibroblast activity - Regulation of tissue remodeling and metabolic pathways	Anti-fibrotic potential, metabolic skin support, wound repair	[90]
Flavonoids	Multiple signaling pathways (Wnt/β-catenin, Hippo, TGF-β, Hedgehog, JNK, NF-κB, MAPK/ERK, PI3K/Akt, Nrf2/ARE)	- Regulate inflammation - Promote angiogenesis - Stimulate reepithelialization - Enhance antioxidant defenses	- Coordinated wound healing responses	Basis for linking *C. zizanioides* activity to broader skin repair mechanisms	[91]

↑ indicates an increase (upregulation), and ↓ indicates a decrease (downregulation) in the corresponding molecule or effect.

**Table 6:** Summary of the mechanisms underlying the anticancer activity of *C. zizanioides*.

Extract Type	Bioactive Compound Involved	Cell Line Model	Effect	References
Essential oil	Cedr-8-en-13-ol, α-/β-pinene, α-/γ-terpinene	B16 melanoma cells	Non-cytotoxic; ↓ melanin, tyrosinase activity, ↑ antioxidant enzymes, ↓ oxidative stress	[98]
Ethanolic root extract	Ellagic acid, Ascorbic acid, Linoleic acid, α-/β-Sitosterol	SCC-29B (oral), DU-145 (prostate), Vero	Cytotoxic to SCC-29B, DU-145; induces DNA damage, apoptosis; minimal Vero toxicity	[53]
*C. zizanioides* acetate oil	Not specified	In vivo: Swiss albino mice treated with cisplatin	Protects from nephrotoxicity, DNA/chromosomal damage; restores GSH/enzymes; ↑ antioxidant defenses	[99]
Essential oil	Sesquiterpene lactones	HeLa (human cervical cancer)	Cytotoxic (IC50 0.05%); induces apoptosis, ROS, mitochondrial depolarization	[100]
Methanol extract (polyherbal including *C. zizanioides*)	Carbohydrates, alkaloids, steroids, saponins, flavonoids, tannins	HeLa, MCF-7	Cytotoxic to HeLa; low toxicity to MCF-7; likely antioxidant mediated	[75]
Ethanolic root extract	Longifolene	DU-145 (prostate cancer), SCC-29B (oral cancer), Vero (healthy kidney)	Cytotoxic to prostate/oral cancer; minimal Vero toxicity	[101]
Aqueous root extract	Valencene	L929 fibroblasts (cytotoxicity)	Cytotoxic; ↑ TNF-α, IL-6; immunomodulatory	[96]
Essential Oil	β-caryophyllene, α-humulene, caryophyllene oxide	In vitro: WiDr (colon), 4T1 (TNBC), T47D (luminal breast) cancer cells; MTT assay	Cytotoxic; ↑ ROS, apoptosis; docking confirms binding	[93]
*C. zizanioides* crude oil and commercial essential oil	Not specified	In vitro: HeLa cervical cancer cells; Mitotic index (MI) assay	Stronger anticancer (MI 1.70%) vs. commercial EO (3.26%) and control (5.57%); ↓ MI → ↑ antimitotic activity; active components not identified.	[94]
*C. zizanioides* oil	Khusimol, aristol-1(10)en-9-ol, cyclocopacamphenol, bicyclo[5.2.0]nonane-2-methylene-4,8,8-trimethyl-4-vinyl	In vitro: Human lung (A549) and hepatocellular (HepG2) cancer cell lines; MTT assay	Moderate cytotoxicity; inhibits proliferation	[92]
*C. zizanioides* oil	Beta vetispirene	In silico: Molecular docking; bioinformatics	Selectively inhibits AKR1C1/2; ↑ ROS, apoptosis (lung cancer)	[95]
Methanolic root extract	Not specified	In vitro: MTT assay on HEK 293 cells	Non-cytotoxic; no viability reduction	[97]

↑ indicates an increase (upregulation), and ↓ indicates a decrease (downregulation) in the corresponding molecule or effect.

## Data Availability

No new data were created or analyzed in this study. Data sharing is not applicable to this article.
